# Structural, Electronic, and Physical Properties of a New Layered Cr-Based Oxyarsenide Sr_2_Cr_2_AsO_3_

**DOI:** 10.3390/ma15030802

**Published:** 2022-01-21

**Authors:** Yi-Qiang Lin, Hao Jiang, Hua-Xun Li, Shi-Jie Song, Si-Qi Wu, Zhi Ren, Guang-Han Cao

**Affiliations:** 1 Department of Physics, Zhejiang University, Hangzhou 310027, China; yiqianglin@zju.edu.cn (Y.-Q.L.); jialay@zju.edu.cn (H.J.); lihuaxun@zju.edu.cn (H.-X.L.); 11936028@zju.edu.cn (S.-J.S.); sqwu@zju.edu.cn (S.-Q.W.); 2 School of Physics and Optoelectronics, Xiangtan University, Xiangtan 411105, China; 3 School of Science, Westlake Institute for Advanced Study, Westlake University, Hangzhou 310064, China; renzhi@westlake.edu.cn; 4 Zhejiang Province Key Laboratory of Quantum Technology and Devices, Interdisciplinary Center for Quantum Information and State Key Lab of Silicon Materials, Zhejiang University, Hangzhou 310027, China

**Keywords:** Cr-based oxyarsenide, physical properties, DFT calculations, antiferromagnetism, interlayer charge transfer

## Abstract

We report synthesis, crystal structure, and physical properties of Sr ${}_{2}$ Cr ${}_{2}$ AsO ${}_{3}$ . The new compound crystallizes in a Sr ${}_{2}$ GaO ${}_{3}$ CuS-type structure with two distinct Cr sites, Cr(1) in the perovskite-like block layers of “Sr ${}_{3}$ Cr ${}_{2}$ O ${}_{6}$ ” and Cr(2) in the ThCr ${}_{2}$ Si ${}_{2}$ -type layers of “SrCr ${}_{2}$ As ${}_{2}$ ”. An inter-block-layer charge transfer is explicitly evidenced, which dopes electrons in the CrO ${}_{2}$ planes and simultaneously dopes holes into the CrAs layers. Measurements of electrical resistivity, magnetization, and specific heat, in combination with density-functional theoretical calculations, indicate that the title material is an antiferromagnetic metal. The Cr(2) magnetic moments in the CrAs layers order at 420 K, while the Cr(1) spins in the CrO ${}_{2}$ planes show quasi-two-dimensional magnetism with long-range ordering below 80 K. Both Néel temperatures are significantly reduced, compared with those of the cousin material Sr ${}_{2}$ Cr ${}_{3}$ As ${}_{2}$ O ${}_{2}$ , probably due to the intrinsic charge-carrier doping. Complex re-entrant magnetic transitions with a huge magnetic hysteresis were observed at low temperatures.

## 1. Introduction

The discoveries of superconductivity in CrAs [1,2] at high pressures and, later in quasi-one-dimensional $A_{2}$ Cr ${}_{3}$ As ${}_{3}$ (*A* = K, Rb, Cs) at ambient pressure [3,4,5], motivate the research interest in other Cr-based materials [6,7,8,9,10,11,12,13,14,15,16]. Of particular interest are those with CrAs layers [17,18,19,20], which resemble the FeAs layers in iron-based superconductors [21]. Similar to the parent compounds of iron-based superconductors, these CrAs-layer based materials, such as BaCr ${}_{2}$ As ${}_{2}$ [18] and LaCrAsO [20], also show antiferromagnetic (AFM) order and metallic conduction. Theoretical studies [6,7,8] suggest possible superconductivity in doped BaCr ${}_{2}$ As ${}_{2}$ and LaCrAsO. Notably, the Néel temperatures are as high as ∼600 K (see Table 1), suggesting a large magnetic exchange interaction. If the AFM order can be suppressed, according to the common features of unconventional superconductivity [22], one expects possible high-temperature superconductivity in the class of Cr-based materials. In this context, explorations for additional materials with CrAs layers are of significance [23,24,25,26].

In 2015, the present authors [23] succeeded in synthesizing a Cr-based oxyarsenide Sr ${}_{2}$ Cr ${}_{3}$ As ${}_{2}$ O ${}_{2}$ . Structurally, the compound can be viewed as an intergrowth of ThCr ${}_{2}$ Si ${}_{2}$ -type SrCr ${}_{2}$ As ${}_{2}$ and perovskite-like infinite-layer “SrCrO ${}_{2}$ ” [27], thus bearing both CrAs layers and CrO ${}_{2}$ planes. The material was demonstrated to be an AFM correlated metal. Later, the neutron diffraction study [28] revealed that the magnetic moments of Cr(1) (in the CrO ${}_{2}$ planes) and Cr(2) (in the CrAs layers) are 3.10(6) $\mu_{B}$ and 2.19(4) $\mu_{B}$ , respectively. The Cr(1) spins form a La ${}_{2}$ CuO ${}_{4}$ -like AFM order at 291 K, and the Cr(2) moments show a C-type (Néel-type antiferromagnetism in the ab plane and ferromagnetism along the *c* axis) AFM order below 590 K.

**Figure 1:**
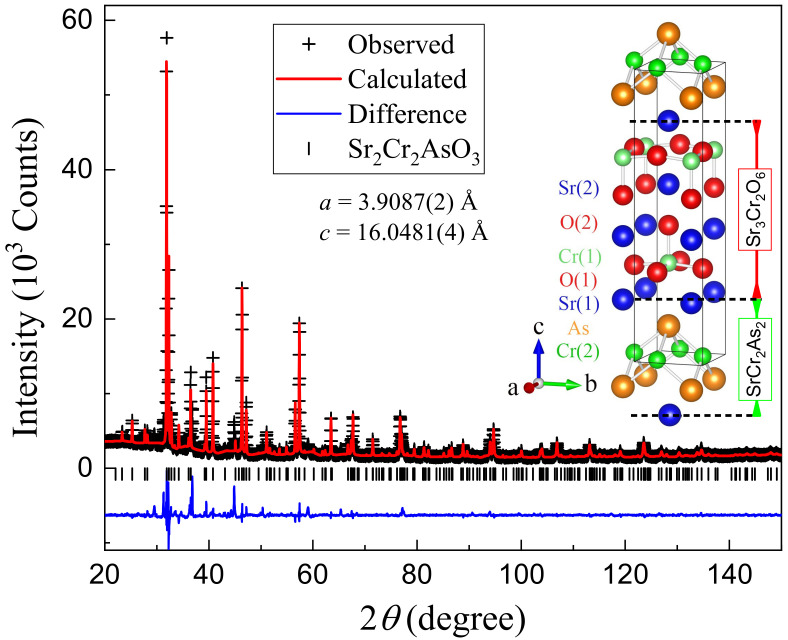
Powder X-ray diffraction at room temperature and its Rietveld refinement profile of Sr ${}_{2}$ Cr ${}_{2}$ AsO ${}_{3}$ . The inset shows the crystal structure consisting of block layers of Sr ${}_{3}$ Cr ${}_{2}$ O ${}_{6}$ and SrCr ${}_{2}$ As ${}_{2}$ .

We note that the perovskite-like block layer $A_{3}$ Cr ${}_{2}$ O ${}_{6}$ ( A= Sr, Ba) can be intergrown with the ThCr ${}_{2}$ Si ${}_{2}$ -type $AM_{2}$ As ${}_{2}$ ( M= Fe, Co) [30,31], forming a Sr ${}_{2}$ GaO ${}_{3}$ CuS-type structure [32]. This suggests the possibility of synthesis of an analogous Cr-based oxyarsenide, Sr ${}_{2}$ Cr ${}_{2}$ AsO ${}_{3}$ , which contains alternating layers of “Sr ${}_{3}$ Cr ${}_{2}$ O ${}_{6}$ ” and “SrCr ${}_{2}$ As ${}_{2}$ ” (see the inset of Figure 1). Notably, the target structure bears distinct Cr valence in different block layers (namely, Cr ${}^{3+}$ in the perovskite-like $A_{3}$ Cr ${}_{2}$ O ${}_{6}$ and Cr ${}^{2+}$ in SrCr ${}_{2}$ As ${}_{2}$ ), in contrast to the case in Sr ${}_{2}$ Cr ${}_{3}$ As ${}_{2}$ O ${}_{2}$ where the valences of Cr(1) and Cr(2) are both 2+. An inter-block-layer charge transfer is thus anticipated, which effectively dopes holes in the CrAs layers. This makes Sr ${}_{2}$ Cr ${}_{2}$ AsO ${}_{3}$ particularly interesting.

In this paper, we report the synthesis, crystal structure, and physical properties of Sr ${}_{2}$ Cr ${}_{2}$ AsO ${}_{3}$ (this phase was identified using electron microscopy as an impurity (1.3%) in the synthesized sample of Sr ${}_{2}$ Cr ${}_{3}$ As ${}_{2}$ O ${}_{2}$ [25]). The crystal structure is determined by the Rietveld refinement, from which the bond-valence sum of Cr(1) is calculated to be +2.52. The result indicates a mixed valence for Cr(1), suggesting an inter-block-layer charge transfer. The charge transfer is also supported by the Cr(1) effective moment from the magnetic measurement and the Bader charge analysis on the basis of density-functional-theory (DFT) calculations. The magnetic measurement reveals that the Néel temperatures in the CrAs layers and the CrO ${}_{2}$ planes are $T_{N}2=$ 420 K and $T_{N}1\approx$ 80 K, which are remarkably reduced, in comparison with the counterparts (∼600 K and ∼300 K, respectively) in Sr ${}_{2}$ Cr ${}_{3}$ As ${}_{2}$ O ${}_{2}$ . The suppression of the Néel temperatures is probably due to the charge-carrier dopings, originating from the interlayer charge transfer. Complex re-entrant magnetic transitions with a huge magnetic hysteresis were observed at low temperatures. The magnetic ground state is studied by the DFT-based first-principles calculations.

## 2. Experimental Methods

The Sr ${}_{2}$ Cr ${}_{2}$ AsO ${}_{3}$ polycrystalline sample was synthesized by solid-state reactions in vacuum. The source materials were Sr pieces (99%), As pieces (99.999%), Cr powders (99.99%), and Cr ${}_{2}$ O ${}_{3}$ powders (99.97%). Mixtures of these raw materials with the nearly nominal composition (the oxygen content is reduced by 6% to compensate the slight oxidation in the reactive Sr pieces) were put into an alumina crucible, sealed in an evacuated silica tube, then calcined in a muffle furnace at 800 ${}^{\circ }$ C for 24 h. After cooling, the sample was thoroughly ground for homogenization and pressed into a pellet in a glove box filled with argon gas. The pellet was also loaded an alumina crucible, sealed in an evacuated silica tube, and then heated to 1100 ${}^{\circ }$ C, holding at this temperature for 40 h. Finally, the sample was allowed to cool down by switching off the furnace.

The X-ray diffraction (XRD) was performed at room temperature using a PANalytical X-ray diffractometer with Cu-K ${}_{\alpha 1}$ radiation. The crystal structure was refined by the Rietveld analysis using FullProf suite [33]. The electrical resistivity and specific heat were measured with a quantum design physical property measurement system (PPMS). The magnetic properties were measured by a quantum design magnetic property measurement system (MPMS-3). The measurement was allowed at temperatures up to 700 K by employing a high-temperature option.

The first-principles calculations were done within the generalized gradient approximation [34] by using the Vienna Ab-initio Simulation Package (VASP) [35]. The experimental crystal structure was used for the calculations. The plane-wave basis energy cutoff was chosen to be 550 eV. A 10 × 10 × 5 Γ -centered K-mesh was used for the density-of-states (DOS) calculations.

## 3. Results and Discussion

### 3.1. Crystal Structure

Figure 1 shows the XRD pattern of the as-prepared polycrystalline sample of Sr ${}_{2}$ Cr ${}_{2}$ AsO ${}_{3}$ . All the strong XRD reflections can be indexed using a primitive tetragonal unit cell with a≈ 3.91 Å and c≈ 16.05 Å. Some unindexed weak peaks probably come from the unreacted CrAs and SrO, while the others cannot be identified with known phases. The lattice constants are very close to those of the analogous compound Sr ${}_{2}$ CrFeAsO ${}_{3}$ [30,31], suggesting the formation of the target phase. Indeed, the XRD data can be successfully refined with the Sr ${}_{2}$ GaO ${}_{3}$ CuS-type structure model [32]. The *R* factors of the refinement are $R_{B}$ = 4.65% and $R_{\mathrm{wp}}$ = 8.21%. Furthermore, the Goodness-of-fit index is 3.8. The refined crystallographic data are listed in Table 2.

As shown in the inset of Figure 1, the crystal structure of Sr ${}_{2}$ Cr ${}_{2}$ AsO ${}_{3}$ can be viewed as an alternating stacking of Sr ${}_{3}$ Cr ${}_{2}$ O ${}_{6}$ and SrCr ${}_{2}$ As ${}_{2}$ block layers along the *c* direction. According to the block-layer model for an intergrowth structure [36], inter-block-layer charge transfer and lattice match are the two major factors for the stabilization. Furthermore, the lattice constant *a* is mainly determined by the structural block with a larger stiffness. In Table 1, one sees that the *a* axis of Sr ${}_{2}$ Cr ${}_{2}$ AsO ${}_{3}$ is close to that of Sr ${}_{2}$ CrFeAsO ${}_{3}$ , but significantly smaller than those of Sr ${}_{2}$ Cr ${}_{3}$ As ${}_{2}$ O ${}_{2}$ and Sr ${}_{2}$ ScCrAsO ${}_{3}$ . The result suggests that the perovskite-like block layers of Sr ${}_{3}$ Cr ${}_{2}$ O ${}_{6}$ , SrCrO ${}_{2}$ , and Sr ${}_{3}$ Sc ${}_{2}$ O ${}_{6}$ are harder, which dominantly determine the *a* axis. Note that the *a* axis of Sr ${}_{2}$ Cr ${}_{2}$ AsO ${}_{3}$ is also close to that of SrCr ${}_{2}$ As ${}_{2}$ , which indicates a very good lattice match between the two building blocks.

There are two distinct crystallographic Cr sites, Cr(1) and Cr(2), in the block layers of Sr ${}_{3}$ Cr ${}_{2}$ O ${}_{6}$ and SrCr ${}_{2}$ As ${}_{2}$ , respectively. The former is pyramidally coordinated by five oxygen atoms with a formal valence of +3, and the latter is bonded tetrahedrally by four As atoms with a formal valence of +2. To evaluate the possible inter-block-layer charge transfer, we calculate the bond valence sum (BVS) [37] of Cr(1) (note that the BVS concept does not apply for Cr(2) because of the dominant covalence of the Cr(2)−As bonding). The calculated BVS value is calculated to be +2.52, suggesting a mixed valence for Cr(1). In comparison, the Cr-BVS value in Sr ${}_{2}$ CrFeAsO ${}_{3}$ is 2.93 [31], and it is 1.9-2.1 in Sr ${}_{2}$ Cr ${}_{3}$ As ${}_{2}$ O ${}_{2}$ [23,25,28]. All these data conformably suggest an interlayer charge transfer between the two block layers, which primarily stabilizes the structure [36]. The interlayer charge transfer leads to electron doping in the CrO ${}_{2}$ planes and hole doping in the CrAs layers. From Table 1, one sees that the As height (above the Cr plane) is exceptionally high and, correspondingly, the As-Cr-As bond angle along *a* axis is exceptionally small. These structural changes may be associated with the effective hole doping in the CrAs layers.

### 3.2. Electrical Resistivity

**Figure 2:**
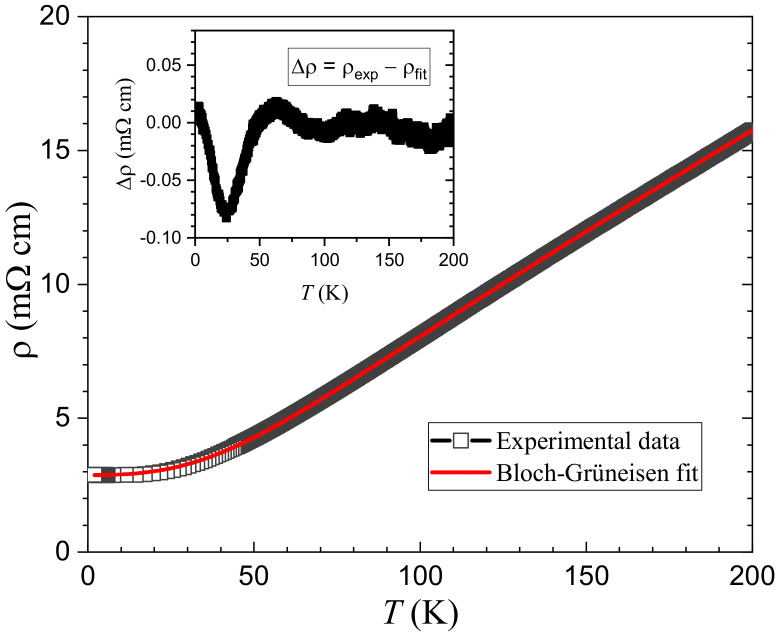
Temperature dependence of electrical resistivity of the Sr ${}_{2}$ Cr ${}_{2}$ AsO ${}_{3}$ polycrystalline sample. The solid line is a fit with the extended Bloch-Grüneisen formula (see the text). The inset shows the difference, Δρ , between the experimental and fitted data. The drop in Δρ by ∼3% below 70 K suggests reduction of the magnetic scattering by Cr(1) spins.

The temperature dependence of resistivity, ρ(T) , for the Sr ${}_{2}$ Cr ${}_{2}$ AsO ${}_{3}$ polycrystalline sample is shown in Figure 2. A typical metallic behavior shows up without any obvious anomaly below room temperature. Similar observations were observed in BaCr ${}_{2}$ As ${}_{2}$ [9,18] and LaCrAsO [20]. For Sr ${}_{2}$ ScCrAsO ${}_{3}$ and Ba ${}_{2}$ ScCrAsO ${}_{3}$ [26], which contain insulating block layers of Sr ${}_{3}$ Sc ${}_{2}$ O ${}_{6}$ and Ba ${}_{3}$ Sc ${}_{2}$ O ${}_{6}$ , respectively, the ρ(T) behaviors are basically metallic. These results suggest that the CrAs layers are mainly responsible for the metallic conduction.

As treated previously [23], the ρ (*T*) data were fitted with the extended Bloch-Grüneisen formula,

(1) $$\rho \left(T\right)=\rho_{0}+A{\left(\frac{T}{\Theta_{R}}\right)}^{n}\int_{0}^{\Theta_{R}/T}\frac{x^{n}}{\left(e^{x}-1\right)\left(1-e^{-x}\right)}dx,$$

where *n* is a fitting parameter characterizing the conduction electron scattering [38,39,40]. The data fitting yields *n* = 2.58, $\Theta_{R}$ = 303 K, and $\rho_{0}$ = 2.87 m Ω cm. One sees that the *n* value lies in between 2 and 3, implying both electron-electron and electron-magnon scattering [39,41]. To reveal the possible change in magnetic scattering, we made the subtraction of fitted data from the experimental ones. The result is plotted in the inset of Figure 2. One sees a broad dip (resistivity decreased by ∼3%) below ∼70 K. This slight response of resistivity could be associated with the magnetic ordering at the Cr(1) site (see below).

### 3.3. Magnetic Properties

**Figure 3:**
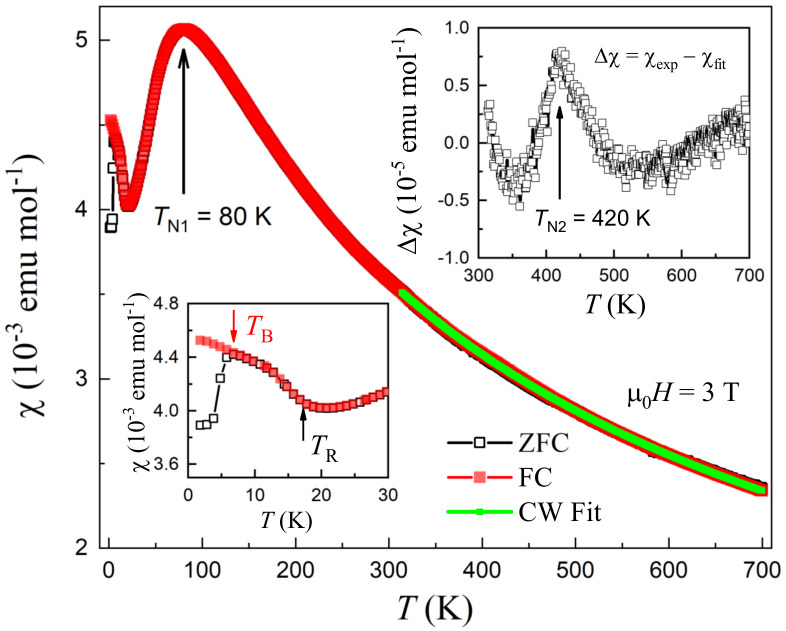
Temperature dependence of magnetic susceptibility, χ(T) , under an applied field of $\mu_{0}H\;=$ 3 T for Sr ${}_{2}$ Cr ${}_{2}$ AsO ${}_{3}$ . The green solid line is the Curie-Weiss (CW) fit. The upper inset plots the difference in χ between the experimental data and the fitted ones. The bottom inset shows the closeup of the χ(T) curves at low temperatures.

Figure 3 shows the temperature dependence of magnetic susceptibility, χ(T) , for Sr ${}_{2}$ Cr ${}_{2}$ AsO ${}_{3}$ . The data show a Curie-Weiss (CW) behavior at high temperatures up to 700 K. The CW behavior is reasonably attributed to the contribution of Cr(1) in the Sr ${}_{3}$ Cr ${}_{2}$ O ${}_{6}$ layers, because the χ(T) of materials with CrAs layers only, such as BaCr ${}_{2}$ As ${}_{2}$ [9], LaCrAsO [20], and Sr ${}_{2}$ ScCrAsO ${}_{3}$ [26], is not CW like. The data fitting in the temperature range from 314 K to 700 K by using the extended CW law, $\chi =\chi_{0}+C/\left(T+\theta_{\mathrm{CW}}\right)$ , yields $\chi_{0}\;=\;3.15\times 10^{-4}$ emu mol ${}^{-1}$ , *C* = 2.13 emu K mol ${}^{-1}$ , and $\theta_{\mathrm{CW}}=352$ K. The positive CW temperature indicates dominant antiferromagnetic interactions among the Cr(1) spins. The fitted parameter *C* gives rises to an effective moment for Cr(1) atoms, $\mu_{\mathrm{eff}}^{\mathrm{Cr}1}=4.13\;\mu {}_{B}$ , which lies in between the expected values of high-spin Cr ${}^{3+}$ (3.87 $\mu {}_{B}$ ) and high-spin Cr ${}^{2+}$ (4.90 $\mu {}_{B}$ ). The result suggests local-moment and mixed-valence scenarios for Cr(1) atoms. The latter arises from the interlayer charge transfer as demonstrated above.

To detect the possible magnetic transition related to the CrAs layers in Sr ${}_{2}$ Cr ${}_{2}$ AsO ${}_{3}$ , we treated the CW magnetism as a background, and made a subtraction of the CW-fit data from the experimental χ(T) data. As is seen in the upper-right inset of Figure 3, a drop of χ shows up at 420 K, indicating an AFM transition. Here we note that the Néel temperature of the Cr(2) sublattice, $T_{N}2$ , is remarkably reduced, compared with those of other CrAs-layer based materials including SrCr ${}_{2}$ As ${}_{2}$ (see Table 1). Since the *a* axes of Sr ${}_{2}$ Cr ${}_{2}$ AsO ${}_{3}$ and SrCr ${}_{2}$ As ${}_{2}$ are comparable, the reduction of $T_{N}2$ in Sr ${}_{2}$ Cr ${}_{2}$ AsO ${}_{3}$ is arguably attributed to the hole-doping effect in the CrAs layers because of the interlayer charge transfer.

Now that the high-temperature CW behavior comes from the Cr(1)-spin paramagnetism, the susceptibility drop below $T_{N}1\approx$ 80 K in Figure 3 is then ascribed to an AFM ordering in Sr ${}_{3}$ Cr ${}_{2}$ O ${}_{6}$ block layers. The slight loss of resistivity below ∼70 K in Figure 2 can be understood in terms of reduction of magnetic scattering. The $T_{N}1$ value is remarkably lower than $\theta_{\mathrm{CW}}=352$ K, implying quasi-two-dimensional (quasi-2D) magnetism. Indeed, there is a broad hump above $T_{N}1$ , reflecting quasi-2D AFM correlations/short-range order [42,43,44,45]. Similar behaviors also appear in Sr ${}_{3}$ Cr ${}_{2}$ O ${}_{6}$ -layer-bearing Sr ${}_{2}$ CrO ${}_{3}$ FeAs [31] and Sr ${}_{2}$ CrO ${}_{3}$ CuS [46]. Besides, the reduced $T_{N}1$ (in comparison with that of Sr ${}_{2}$ Cr ${}_{3}$ As ${}_{2}$ O ${}_{2}$ [25,28]) may be accounted for by the electron doping in the CrO ${}_{2}$ planes.

**Figure 4:**
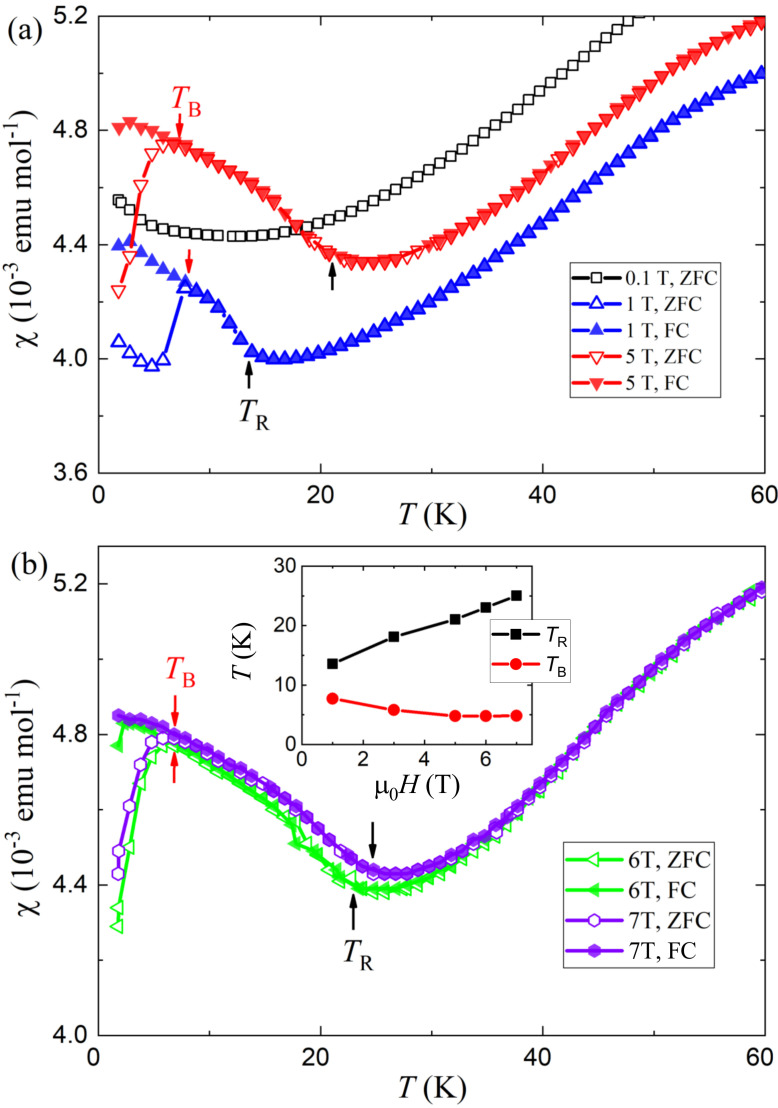
Temperature dependence of magnetic susceptibility for Sr ${}_{2}$ Cr ${}_{2}$ AsO ${}_{3}$ at varied magnetic fields of 0.1, 1, 5 (**a**), 6, and 7 T (**b**) in the field-cooling (FC) and zero-field-cooling (ZFC) measurement modes. The inset of (**b**) shows the changes of $T_{R}$ and $T_{B}$ with the magnetic field.

Interestingly, there exist additional magnetic anomalies at lower temperatures, which are shown in the bottom-left inset of Figure 3. More data at varied magnetic fields are given in Figure 4. One sees that, below $T_{N}1$ , the magnetic susceptibility increases steeply with decreasing temperature at $T_{R}$ , suggesting a re-entrant magnetic transition. With further decreasing temperature, the FC and ZFC data bifurcates at $T_{B}$ , and $\chi_{\mathrm{ZFC}}$ drops abruptly below $T_{B}$ . The inset of Figure 4b plots $T_{R}$ and $T_{B}$ as functions of magnetic field. It is clear that $T_{R}$ increases steadily with field, while $T_{B}$ decreases slowly.

**Figure 5:**
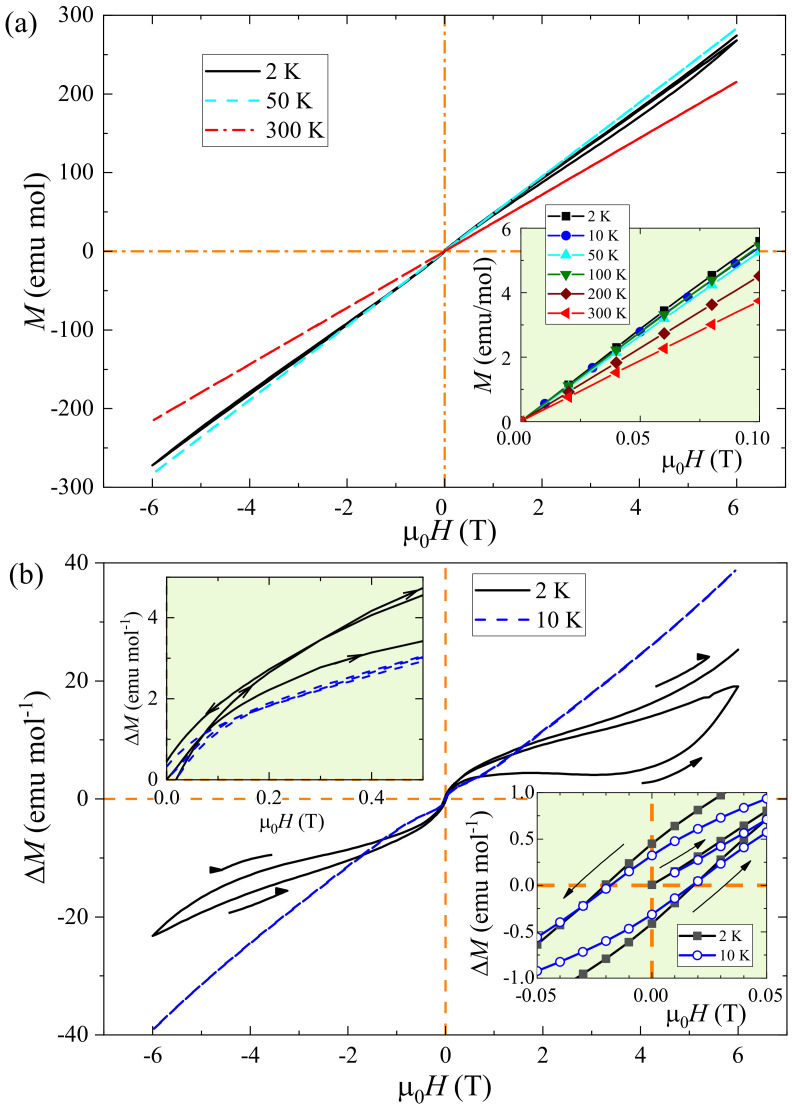
(**a**) Field dependence of magnetization at some selective temperatures for Sr ${}_{2}$ Cr ${}_{2}$ AsO ${}_{3}$ . Panel (**b**) shows the magnetization difference, ΔM=M−41.5H , at 2 and 10 K. All the insets are closeups for showing the details clearly.

In order to clarify the magnetic anomalies above, we carried out the measurements of isothermal magnetization, M(H) , by sweeping magnetic fields in a loop (i.e., $\mu_{0}H\;=\;0\;T\to 6\;T\to -6\;T\to 6\;T$ ). Figure 5 displays some selective M(H) data. At first sight, *M* seems to be linearly proportional to *H* at varied temperatures from 2 K to 300 K. On closer examination, however, a slight magnetic hysteresis can be found. To extract the hysteresis, we made a subtraction, $\Delta M=M-M_{\mathrm{ref}}$ , by employing a paramagnetic reference of $M_{\mathrm{ref}}$ = 41.5 *H*. As a result, an anomalous hysteresis shows up in the ΔM plot [Figure 5b]. In the low-field region, one sees a ferromagnetic-like magnetization with a coercive field of about 0.018 T (180 Oe). This reflects that the re-entrant magnetic transition at $T_{R}$ induces a ferromagnetic component. Nevertheless, the “saturation” magnetization is only about 5 emu/mol, equivalent to ∼10 ${}^{-3}\mu_{B}$ per formula unit. Given the antiferromagnetic states in both Cr(2) and Cr(1) sublattices, then, the small-moment ferromagnetism is likely originated from the Cr-spin canting. The origin of the spin canting could be in relation with double exchange, as shown in the classical literature by de Gennes [47]. Notably, the magnetic hysteresis is huge, consistent with the bifurcation of magnetic susceptibility in FC and ZFC measurements under magnetic fields up to $\mu_{0}H=$ 7 T. Furthermore, the magnetic hysteresis does not form a loop when increasing field from −6 to 6 T. This magnetic behavior is rarely seen. We conjecture that it is due to the complex magnetic structure and, in particular, in relation with the proximity of magnetic energy for different spin directions (see the DFT-based calculations below).

### 3.4. Specific Heat

**Figure 6:**
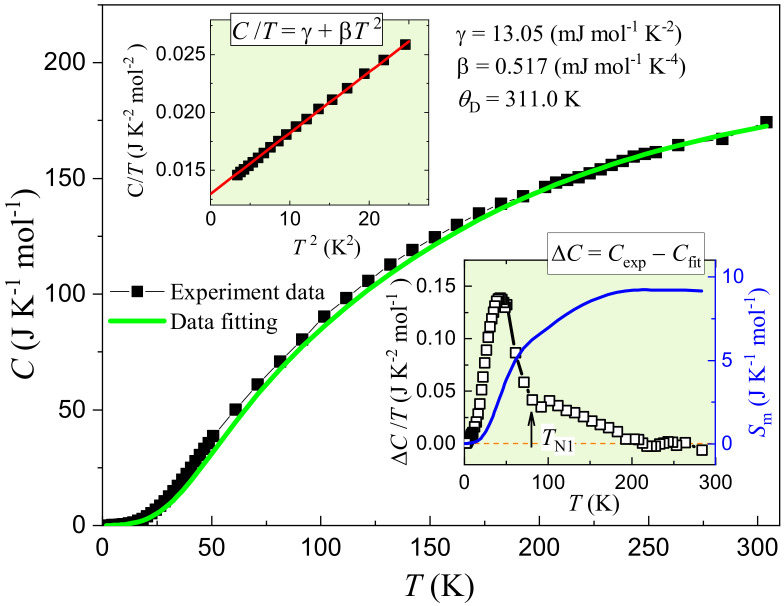
Temperature dependence of specific heat for Sr ${}_{2}$ Cr ${}_{2}$ AsO ${}_{3}$ . The upper inset shows the low-temperature specific-heat data, plotted with C/T vs. $T^{2}$ . The lower inset plots ΔC/T (left axis) and the magnetic entropy (right axis) as functions of temperature (see text for the details).

Figure 6 shows the temperature dependence of specific heat, C(T) , for Sr ${}_{2}$ Cr ${}_{2}$ AsO ${}_{3}$ . At first sight, there is no obvious anomaly below room temperature. That is to say, the AFM transition below 80 K, manifested by the magnetic measurement above, is not associated with an abrupt change in entropy. This is actually consistent with the quasi-2D magnetism, in which magnetic correlations and short-range magnetic order exist at a wide temperature range around $T_{N}1$ .

Let us first analyze the low-temperature C(T) data. At such low temperatures below 5 K, the lattice contribution well obeys Debye’s $T^{3}$ law. Furthermore, the magnetic contributions can be ignored because $T\ll T_{N}$ . Therefore, the total specific heat can be formulated with $C\left(T\right)=\gamma T+\beta T^{3}$ , where γ is the electronic specific-heat coefficient. The upper inset of Figure 6 plots C/T as a function of $T^{2}$ in the low temperature range from 2 to 5 K. Indeed, one sees that C/T is essentially linear with $T^{2}$ . The linear fitting gives γ = 13.05 mJ K ${}^{-2}$ mol ${}^{-1}$ and β = 0.517 mJ K ${}^{-4}$ mol ${}^{-1}$ . Using the formula $\Theta_{D}={\left[\left(12/5\right)NR\pi^{4}/\beta \right]}^{1/3}$ , we obtain a Debye temperature of 311.0 K. The $\Theta_{D}$ value is rather close to $\Theta_{R}$ obtained from the fit of resistivity above.

Now we analyze the whole C(T) data, in order to extract the possible magnetic contribution from Cr(1) spins. Here, the magnetic contribution from Cr(2) can be ignored, because $T_{N}2$ is well above room temperature. Then, similar to our previous treatment [23], the non-magnetic contribution of specific heat can be expressed by:

(2) $$C=\xi C_{D}+\left(1-\xi \right)C_{E}+\gamma T,$$

where $C_{D}=9NR{\left(\frac{T}{\Theta_{D}}\right)}^{3}$ $\int_{0}^{\Theta_{D}/T}\frac{x^{4}e^{x}}{{\left(e^{x}-1\right)}^{2}}dx$ and $C_{E}=3NR{\left(\frac{\Theta_{E}/T}{e^{\Theta_{E}/T}}\right)}^{2}e^{\Theta_{E}/T}$ represent the lattice contributions in terms of Debye and Einstein models, respectively. The coefficients, ξ and ( 1−ξ ), describe their individual weights. With γ and $\Theta_{D}$ fixed (13.05 mJ K ${}^{-2}$ mol ${}^{-1}$ and 311.0 K, respectively), only two adjust parameters, ξ and $\Theta_{E}$ , were fitted. The data in the range of 10≤T≤200 K were dropped in the fitting, because the magnetic contribution cannot be ignored there. As a result, $\Theta_{E}$ = 668.7 K and ξ= 0.616 were yielded. The high $\Theta_{E}$ value is consistent with the lattice vibrations associated with light (oxygen) atoms and, accordingly, the fitted ξ value is very close to the fraction of heavy-element atoms (5/8).

With the fitted data $C_{\mathrm{fit}}$ , the magnetic contribution from Cr(1) spins can be evaluated by the subtraction, $C_{m}^{\mathrm{Cr}1}\approx \Delta C=C_{\exp}-C_{\mathrm{fit}}$ . The lower inset of Figure 6 plots the temperature dependence of the resulted ΔC/T . The main peak appears below $T_{N}1=$ 80 K, which is consistent with the magnetic susceptibility measurement. One also sees a long tail extending to ∼200 K, indicative of magnetic contributions well above $T_{N}1$ . This corroborates quasi-2D magnetic correlations/short-range order in the system. The magnetic entropy, $S_{m}$ , can be obtained by the integral $\int_{0}^{T}\frac{C_{m}}{T}dT$ , and the resulted $S_{m}$ is 9.1 J K ${}^{-1}$ mol ${}^{-1}$ , which is slightly smaller, yet reasonably close to, the expected value of *R*ln (2S+1)= 11.5 J K ${}^{-1}$ mol ${}^{-1}$ (here S= 3/2 is taken). The result is supported by the theoretical calculations below which shows an ordered moment of 2.52 $\mu_{B}$ for Cr(1).

### 3.5. First-Principles Calculations

The possible magnetic ground state of Sr ${}_{2}$ Cr ${}_{2}$ AsO ${}_{3}$ was investigated by the DFT-based first-principles calculations. Owing to the complicated crystal structure with both CrO ${}_{2}$ planes and CrAs layers, we did not calculate all the combinations of Cr-spin orderings. In stead, we took two steps to find out the possible magnetic ground state. First, we focused on the magnetic structure associated with Cr(2) moments, assuming that Cr(1) is non-spin-polarized. This is because the magnetic exchange interactions in the Cr(2) subsystem are expected to be larger due to the higher $T_{N2}$ value and, indeed, there is a temperature window which shows AFM order for Cr(2) and paramagnetism for Cr(1) [25,28]. Second, the whole magnetic structure was optimized by switching on the local spins of Cr(1). The spin directions were chosen along the crystallographic axes, according to the experimental results of the related compounds [9,11,20,25,28,29].

Table 3 lists the Cr moments as well as the magnetic energy $E_{m}$ relative to the non-magnetic state for different magnetic structures of Sr ${}_{2}$ Cr ${}_{2}$ AsO ${}_{3}$ . The first six rows show the results with non-magnetic Cr(1). Clearly, the in-plane Néel-type antiferromagnetism in the Cr(2) sublattice shows the lowest magnetic energy. Cr spins tend to align along the *c* axis. Furthermore, G- and C-type AFM configurations have almost the same magnetic energy (within the calculation accuracy), indicating a very weak interlayer magnetic coupling and, therefore, both magnetic structures are possible. Experimentally, the G-type AFM order exists in BaCr ${}_{2}$ As ${}_{2}$ [9], SrCr ${}_{2}$ As ${}_{2}$ [11], EuCr ${}_{2}$ As ${}_{2}$ [29], and LaCrAsO [20]. Nevertheless, the C-type AFM order is manifested by Sr ${}_{2}$ Cr ${}_{3}$ As ${}_{2}$ O ${}_{2}$ [25,28] and Ba ${}_{2}$ Cr ${}_{3}$ As ${}_{2}$ O ${}_{2}$ [25] in which the distance between CrAs layers is elongated. We thus anticipate that the C-type order is more likely in Sr ${}_{2}$ Cr ${}_{2}$ AsO ${}_{3}$ .

**Figure 7:**
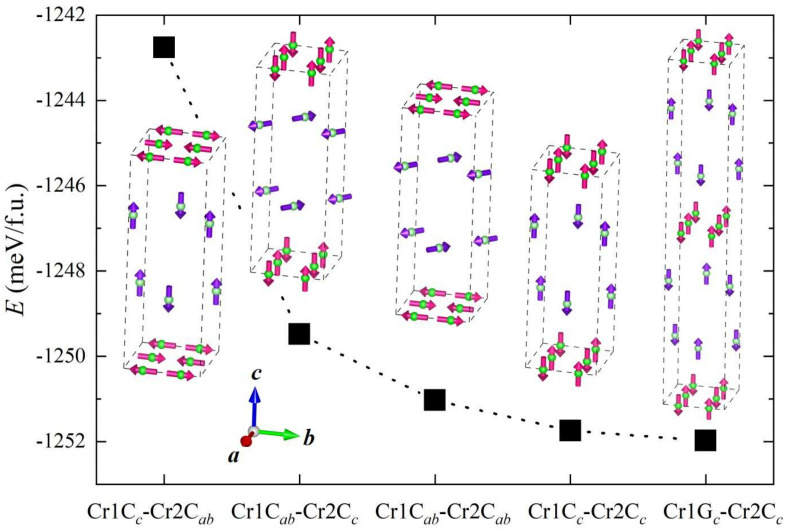
Calculated magnetic energies for the antiferromagnetic states with different spin directions in Cr(1) (**top** and **bottom**) and Cr(2) (**middle**) sites of Sr ${}_{2}$ Cr ${}_{2}$ AsO ${}_{3}$ .

The last nine rows in Table 3 present the calculated data for magnetic structures with the C-type magnetic order in CrAs layers. One can find that the Cr(1) spins also favor an in-plane Néel-type antiferromagnetism with collinear configuration. The Cr-spin direction parallel to *c* axis is mostly stabilized. Nevertheless, the G-type spin order is slightly favored over the C-type one, although the energy difference is quite small. Note that a similar G-type order appears in Sr ${}_{3}$ Cr ${}_{2}$ O ${}_{7}$ with CrO ${}_{2}$ bilayers [48]. Figure 7 depicts the variations of magnetic energy for different magnetic configurations. The most probable magnetic ground state is predicted to be a G/C-type AFM for Cr(1) and a C-type AFM for Cr(2) with spins along the *c* axis. The prediction resembles the experimental results in Sr ${}_{2}$ Cr ${}_{3}$ As ${}_{2}$ O ${}_{2}$ [25,28] and Ba ${}_{2}$ Cr ${}_{3}$ As ${}_{2}$ O ${}_{2}$ [25].

The ordered moments of Cr(1) and Cr(2) are calculated to be 2.52 and 2.41 $\mu_{B}$ , respectively. One notes that the magnetic moment of Cr(1) is reduced by ∼0.5 $\mu_{B}$ , compared with the Cr(1) moment of 3.10 $\mu_{B}$ (experimental value [28]) and 2.97 $\mu_{B}$ (theoretical result [23]) in Sr ${}_{2}$ Cr ${}_{3}$ As ${}_{2}$ O ${}_{2}$ . In the ionic limit, the Cr(1) moment in the high-spin state would be $\mu_{\mathrm{Cr}}=gS=$ 4 (3) $\mu_{B}$ for Cr ${}^{2+}$ (Cr ${}^{3+}$ ) in Sr ${}_{2}$ Cr ${}_{3}$ As ${}_{2}$ O ${}_{2}$ (Sr ${}_{2}$ Cr ${}_{2}$ AsO ${}_{3}$ ), if there were no interlayer charge transfer. The reduction of Cr(1) moment by ∼0.5 $\mu_{B}$ dictates the mixed-valence state as demonstrated above.

To evaluate the charge transfer further, we also performed calculations of the Bader valence charges for different elements in Sr ${}_{2}$ Cr ${}_{3}$ As ${}_{2}$ O ${}_{2}$ and Sr ${}_{2}$ Cr ${}_{2}$ AsO ${}_{3}$ . The results are presented in Table 4. First, the cations of Sr and Cr have less charges, while the anions of As and O have more charges, compared with corresponding neutral atoms. Second, the Bader charges of the cations (anions) decrease (increase) from Sr ${}_{2}$ Cr ${}_{3}$ As ${}_{2}$ O ${}_{2}$ to Sr ${}_{2}$ Cr ${}_{2}$ AsO ${}_{3}$ . In particular, the Bader charge of Cr(1) decreases by 0.6, suggesting a mixed-valence state for Cr(1). On the other hand, the Bader charge of Cr(2) also decreases significantly, consistent with a hole doping in the CrAs layers.

**Figure 8:**
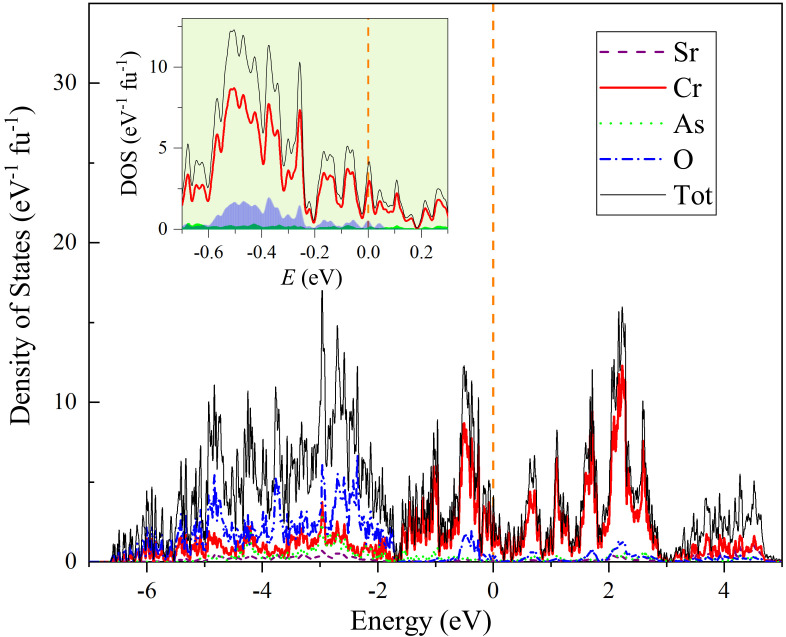
Energy dependence of total and partial electronic density of states (DOS) of the C-type antiferromagnetic state of Sr ${}_{2}$ Cr ${}_{2}$ AsO ${}_{3}$ .

Figure 8 shows the energy dependence of electronic density of states (DOS) for the C-type AFM state in Sr ${}_{2}$ Cr ${}_{2}$ AsO ${}_{3}$ . Both the projected DOS to each element and the total DOS are presented. One sees that the Cr atoms dominantly contribute the DOS at around the Fermi energy ( $E_{F}$ ). Meanwhile, the hybridizations between Cr-3*d* and O-2*p* and between Cr-3*d* and As-4*p* are not negligible (O-2*p* and As-4*p* contribute 12% and 3% of the total DOS, respectively). Overall, the DOS profile is quite similar to that of Sr ${}_{2}$ Cr ${}_{3}$ As ${}_{2}$ O ${}_{2}$ [23]. The bare DOS at the Fermi level is $N(E_{F})$ = 4.08 eV ${}^{-1}$ f.u. ${}^{-1}$ based on both spin directions. With the formula of $\gamma_{0}=\frac{1}{3}\pi^{2}k_{B}^{2}N\left(E_{F}\right)$ , we get $\gamma_{0}$ = 11.3 mJ K ${}^{-2}$ mol ${}^{-1}$ . The $\gamma_{0}$ value is close to the electronic specific-heat coefficient above ( γ = 13.05 mJ K ${}^{-2}$ mol ${}^{-1}$ ), suggesting relatively weak electron correlations.

**Figure 9:**
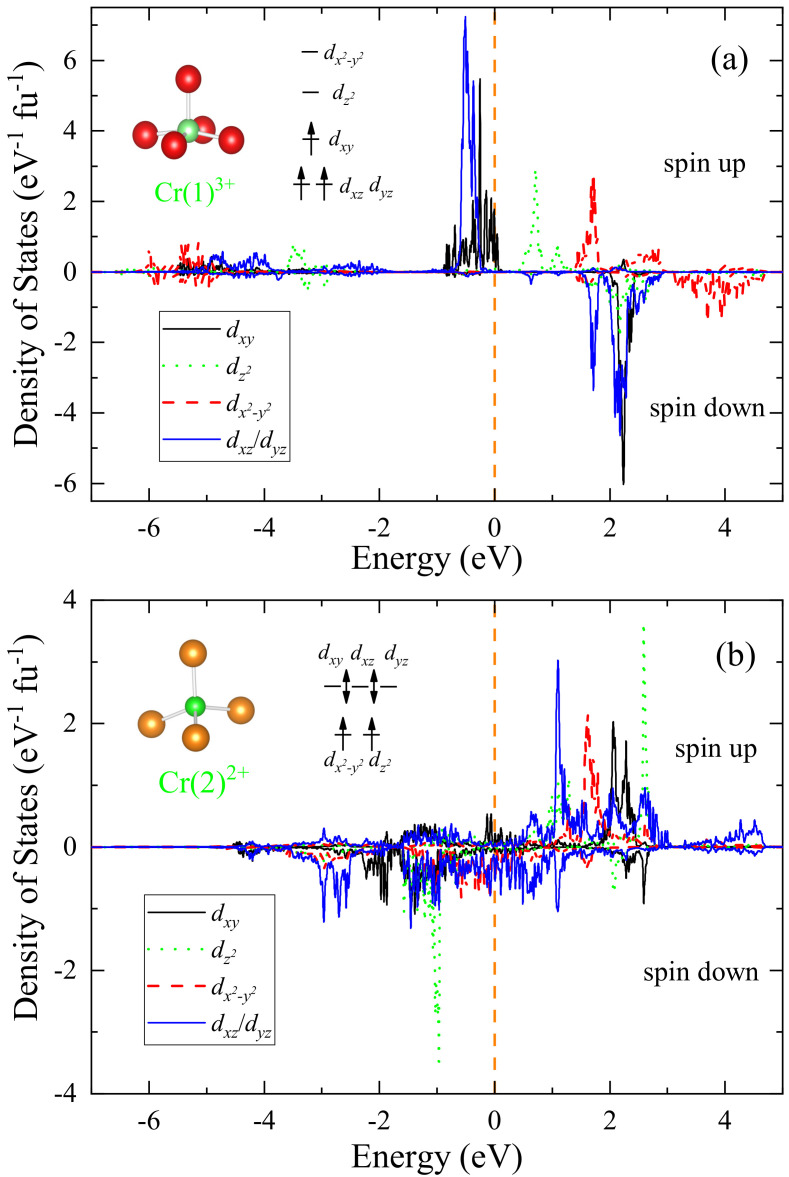
Projected density of states of 3*d* orbitals of the Cr(1) at (1/4, 1/4, 0.3116) (**a**) and Cr(2) at (1/4, 3/4, 0) (**b**) sites in the C-type antiferromagnetic state of Sr ${}_{2}$ Cr ${}_{2}$ AsO ${}_{3}$ . The insets show the coordinations of Cr(1) and Cr(2) and their corresponding crystalline field splittings.

As the Cr-3*d* electrons dominate the states at around $E_{F}$ , the DOS was then projected to each 3d orbitals. Figure 9 shows the projected DOS with spin up (positive DOS) and spin down (negative DOS). The contributions of $d_{xz}$ and $d_{yz}$ orbitals are almost degenerate and, for simplicity, the data are combined. For Cr(1) in the CrO ${}_{2}$ planes, $d_{x^{2}-y^{2}}$ and $d_{z^{2}}$ orbitals are almost unoccupied (the DOS weights below −2 eV are due to the d−p hybridizations). By contrast, the $d_{yz/xz}$ and $d_{xy}$ orbitals are almost single occupied, which gives rise to a magnetic moment close to 3 $\mu_{B}$ . The projected DOS at $E_{F}$ is mostly contributed from $d_{xy}$ orbital. The result can be roughly understood in terms of ionic crystalline electric field (CEF) splitting. Cr(1) ions are coordinated by five oxygen anions, forming a square pyramid. Under this CEF, the $d_{x^{2}-y^{2}}$ and $d_{z^{2}}$ orbitals are lifted up, thus no electrons fill the two orbitals. On the other hand, the other three orbitals are half filled (see the inset of Figure 9a). One sees that these $d_{xy/yz/xz}$ -orbital-related bands are very narrow, suggesting that the Cr(1) magnetism is basically localized.

The Cr(2) atoms in the CrAs layers are coordinated by four arsenic atoms, forming a tetrahedron. The CEF splitting leads to the lift for $d_{xy/yz/xz}$ orbitals. Nevertheless, these three orbitals strongly hybridize with As-4*p* electrons, which makes the orbitals effectively occupied with less spin polarizations. On the other hand, the $d_{x^{2}-y^{2}}$ and $d_{z^{2}}$ appear to be basically half filled, which predominately contributes the magnetic moments. The projected DOS at $E_{F}$ for Cr(2) mostly comes from the $d_{x^{2}-y^{2}}$ and $d_{yz/xz}$ orbitals. Like the case in other CrAs-layer based materials, the magnetism in the CrAs layers is largely itinerant.

## 4. Concluding Remarks

In summary, we have synthesized a novel Cr-based oxyarsenide Sr ${}_{2}$ Cr ${}_{2}$ AsO ${}_{3}$ , which is comprehensively studied by the structural determination, physical-property measurements, and the first-principles calculations. The new material is an intergrowth of perovskite-like “Sr ${}_{3}$ Cr ${}_{2}$ O ${}_{6}$ ” and ThCr ${}_{2}$ Si ${}_{2}$ -type SrCr ${}_{2}$ As ${}_{2}$ , containing both CrO ${}_{2}$ planes and CrAs layers. Thus, like the cousin compound Sr ${}_{2}$ Cr ${}_{3}$ As ${}_{2}$ O ${}_{2}$ , the material bears structural similarities with cuprate and iron-based high-temperature superconductors. Of particular interest in the material is that the Cr atoms in the two block layers show different formal valences. As a result, an inter-block-layer charge transfer is realized, which effectively dopes holes (electrons) in the CrAs (CrO ${}_{2}$ ) layers. Such a charge-carrier doping is argued to be the main cause for the reduced $T_{N}2$ and $T_{N}1$ . If the AFM Néel temperature in the CrAs layers can be further suppressed by chemical doping and/or applying high pressures, one would expect realization of superconductivity in the system according to the common thread of unconventional superconductors [22].

The title material is demonstrated to be an AFM metal with relatively weak electron correlations. While its CrAs layers show an AFM order at $T_{N}2=$ 420 K, the CrO ${}_{2}$ planes exhibit a quasi-2D magnetism with long-range spin ordering below $T_{N}2=$ 80 K. At low temperatures, a re-entrant magnetic transitions with a huge magnetic hysteresis shows up, reflecting complex interactions among Cr(1) and Cr(2) spins and conduction electrons. Our first-principles calculations predict C-type AFM order in the CrAs layers together with C- or G-type AFM order in the CrO ${}_{2}$ planes. Future study with neutron diffraction is called for to verify the magnetic structure and to clarify the re-entrant magnetic transition.

## Figures and Tables

**Table 1:** Comparison of structural (at room temperature) and physical properties of the compounds with CrAs layers and/or CrO ${}_{2}$ planes. $h_{\mathrm{CrAs}}$ denotes the As height from the Cr plane. $\alpha_{\mathrm{As}-\mathrm{Cr}-\mathrm{As}}$ is the As-Cr-As bond angle along *a* or *b* direction. BVS is the bond valence sum of Cr(1) coordinated by five oxygen atoms. $T_{N1}$ and $T_{N2}$ are the antiferromagnetic transition temperatures associated with Cr(1) and Cr(2), respectively, and $\mu_{\mathrm{Cr}1}$ and $\mu_{\mathrm{Cr}2}$ are their saturation magnetic moments. The symbol “-” represents that those data are not available.

Compounds	*a* (Å)	*c* (Å)	c/a	$h_{\mathrm{CrAs}}$ (Å)	$\alpha_{\mathrm{As}-\mathrm{Cr}-\mathrm{As}}$ ( ${}^{\circ }$ )	BVS	$T_{N1}$ (K)	$\mu_{\mathrm{Cr}1}$ ( $\mu_{B}$ )	$T_{N2}$ (K)	$\mu_{\mathrm{Cr}2}$ ( $\mu_{B}$ )	Refs.
BaCr ${}_{2}$ As ${}_{2}$	3.9678(4)	13.632(3)	3.436	1.513	105.3	-	-	-	-	-	[18]
BaCr ${}_{2}$ As ${}_{2}$	3.9667	13.6214	3.434	1.512	105.4	-	-	-	580	1.9	[9]
SrCr ${}_{2}$ As ${}_{2}$	3.918(3)	13.05(1)	3.331	1.449	107.0	-	-	-	590 [11]	1.9(1)	[17]
EuCr ${}_{2}$ As ${}_{2}$	3.893(2)	12.872(2)	3.306	1.455	106.5	-	-	-	680 [29]	1.7(4)	[19]
LaCrAsO	4.0412(3)	8.9863(7)	2.224	1.460	108.3	-	-	-	>300	-	[20]
Sr ${}_{2}$ Cr ${}_{3}$ As ${}_{2}$ O ${}_{2}$	4.0079(1)	18.8298(3)	4.698	1.505	106.2	2.10	291	2.97 (calc.)	-	2.59 (calc.)	[27]
Sr ${}_{2}$ Cr ${}_{3}$ As ${}_{2}$ O ${}_{2}$	4.00671(6)	18.8310(5)	4.700	1.503	106.2	1.90	291	3.10(6)	590	2.19(4)	[28]
Sr ${}_{2}$ Cr ${}_{3}$ As ${}_{2}$ O ${}_{2}$	4.00800(2)	18.8214(1)	4.696	1.505	106.4	1.90	330	3.34(1)	600	2.68(1)	[25]
Ba ${}_{2}$ Cr ${}_{3}$ As ${}_{2}$ O ${}_{2}$	4.05506(2)	20.5637(1)	5.071	1.478	107.8	1.79	230	3.30(1)	465	2.23(1)	[25]
Sr ${}_{2}$ CrFeAsO ${}_{3}$	3.9112(1)	15.7905(3)	4.037	-	-	2.69	31	-	-	-	[30]
Sr ${}_{2}$ CrFeAsO ${}_{3}$	3.918	15.683	4.003	-	-	2.93	-	-	-	-	[31]
Sr ${}_{2}$ ScCrAsO ${}_{3}$	4.043(9)	16.038(1)	3.967	1.498	106.9	-	-	-	-	-	[26]
Sr ${}_{2}$ Cr ${}_{2}$ AsO ${}_{3}$	3.9087(2)	16.0481(4)	4.300	1.560	102.8	2.52	80	2.52 (calc.)	420	2.41 (calc.)	This work

**Table 2:** Room-temperature crystallographic data of Sr ${}_{2}$ Cr ${}_{2}$ AsO ${}_{3}$ with lattice parameters of *a* = 3.9087(2) Å and *c* = 16.0481(4) Å, and with space group *P*4/ nmm (No. 129).

Atoms	Sites	*x*	*y*	*z*	*B*(Å ${}^{2}$ )
Sr(1)	2*c*	0.75	0.75	0.1997(3)	0.56
Sr(2)	2*c*	0.75	0.75	0.4177(3)	0.90
Cr(1)	2*c*	0.25	0.25	0.3117(4)	0.81
Cr(2)	2*a*	0.25	0.75	0	0.61
As	2*c*	0.25	0.25	0.0973(3)	0.36
O(1)	4*f*	0.25	0.75	0.2945(10)	1.01
O(2)	2*c*	0.25	0.25	0.4411(16)	0.73

**Table 3:** Calculated magnetic energy $E_{m}$ (in meV/f.u.) relative to non-magnetic (N) state and the corresponding magnetic moment $\mu_{\mathrm{Cr}}$ (in $\mu_{B}$ ) of different magnetic structures of Sr ${}_{2}$ Cr ${}_{2}$ AsO ${}_{3}$ . F, S, C, and G denote ferromagnetic, striped AFM, C-type AFM, and G-type AFM structures, respectively. The subscripts ab and *c* tell the spin directions.

Magnetic Structure	$E_{m}$	$\mu_{\mathrm{Cr}(1)}$	$\mu_{\mathrm{Cr}(2)}$
Cr(1)N−Cr(2)N	0	0	0
Cr(1)N−Cr(2)F	−67.9	0	1.726
Cr(1)N−Cr(2)S	−0.5	0	0.078
Cr(1)N−Cr(2)C ${}_{ab}$	−310.9	0	2.400
Cr(1)N−Cr(2)C ${}_{c}$	−311.3	0	2.401
Cr(1)N−Cr(2)G ${}_{c}$	−311.1	0	2.401
Cr(1)F−Cr(2)C ${}_{c}$	−911.2	2.653	0.013
Cr(1)F−Cr(2)C ${}_{ab}$	−911.2	2.653	0.013
Cr(1)S−Cr(2)C ${}_{c}$	−987.3	2.590	1.085
Cr(1)S−Cr(2)C ${}_{ab}$	−1144.4	2.206	0.567
Cr(1)C ${}_{c}-$ Cr(2)C ${}_{ab}$	−1242.8	2.529	2.417
Cr(1)C ${}_{ab}-$ Cr(2)C ${}_{c}$	−1249.5	2.521	2.407
Cr(1)C ${}_{ab}-$ Cr(2)C ${}_{ab}$	−1251.0	2.522	2.408
Cr(1)C ${}_{c}-$ Cr(2)C ${}_{c}$	−1251.7	2.522	2.409
Cr(1)G ${}_{c}-$ Cr(2)C ${}_{c}$	−1252.0	2.522	2.409

**Table 4:** Bader charges of different elements in the magnetic ground states of Sr ${}_{2}$ Cr ${}_{2}$ AsO ${}_{3}$ (2213) and Sr ${}_{2}$ Cr ${}_{3}$ As ${}_{3}$ O ${}_{2}$ (2322).

	Bader Charge			
Element	$Q_{2213}$	$Q_{2322}$	$Q_{2213}-Q_{2322}$	Neutral
Sr	8.518	8.706	−0.188	10
Cr(1)	10.407	11.011	−0.604	12
Cr(2)	11.396	11.619	−0.223	12
As	16.282	16.012	0.27	15
O	7.300	7.157	0.143	6

## Data Availability

The datasets used and/or analyzed during the current study are available from the corresponding author on reasonable request.
